# Editorial: Prognostic imaging biomarkers in psychotic disorders

**DOI:** 10.3389/fpsyt.2022.1053836

**Published:** 2022-10-17

**Authors:** Daiki Sasabayashi, Shinsuke Koike, Shinichiro Nakajima, Yoji Hirano

**Affiliations:** ^1^Department of Neuropsychiatry, University of Toyama Graduate School of Medicine and Pharmaceutical Sciences, Toyama, Japan; ^2^Research Center for Idling Brain Science, University of Toyama, Toyama, Japan; ^3^The University of Tokyo Institute for Diversity and Adaptation of Human Mind, Tokyo, Japan; ^4^Center for Evolutionary Cognitive Sciences, Graduate School of Art and Sciences, The University of Tokyo, Tokyo, Japan; ^5^Department of Neuropsychiatry, Keio University School of Medicine, Tokyo, Japan; ^6^Brain Health Imaging Centre, Centre for Addiction and Mental Health, Toronto, ON, Canada; ^7^Department of Neuropsychiatry, Kyushu University, Fukuoka, Japan; ^8^Neural Dynamics Laboratory, Research Service, Department of Psychiatry, VA Boston Healthcare System, Harvard Medical School, Boston, MA, United States

**Keywords:** prognosis, biomarker, outcome, schizophrenia, psychotic disorder, early detection

Psychotic disorders, including schizophrenia, predominantly occur in late adolescence and early adulthood and can affect social functioning of patients throughout their lifetime (1, 2). Given that patients with psychotic disorders exhibit diverse clinical courses, such as their clinical symptoms easily disappearing, fluctuating, or persisting without treatment response (3), psychotic disorders may be not a unitary disease but rather a heterogeneous collection of syndromes. Substantial biological research in psychotic disorders using molecular, neurophysiological, and neuroimaging measurements has demonstrated that neurobiological abnormalities are likely present at illness onset and before onset (4–10), and that distinct patterns of neurobiological changes are mixed within psychotic disorders (11, 12). Such neurobiological heterogeneity may represent different subsequent clinical courses and functional outcomes (2, 13–16).

Comprehensive early intervention services on first-episode psychosis and high-risk state for psychosis have been reported to lead to better clinical and functional outcomes (17, 18), possibly preventing or ameliorating active brain changes during the critical period (5). On the other hand, the early intervention paradigm still needs to be improved, as a recent study reported that the advantages of early intervention services over other interventions in terms of their effects on outcomes do not persist over the long term (19). Furthermore, an early intervention based on biomarkers is needed to prevent the patient from transitioning to treatment-resistant schizophrenia (20). An important strategy to further upgrade early intervention quality is utilizing biomarkers that can predict the outcome. Specifically, stratifying patients based on objective indicators at an early stage of psychosis and providing specific treatment as needed may improve their long-term prognosis (21). However, few studies have examined the relationship between prognosis and neurobiological characteristics in psychotic disorders, and no clinically reliable biomarkers have yet been identified. Therefore, this special issue on “*Prognostic Imaging Biomarkers in Psychotic Disorders*” requested novel findings regarding those relationships, especially in early psychosis.

The six studies (one mini-review and five original investigations) were collected to assess neurobiological characteristics at baseline in association with clinical, cognitive, and functional outcomes in psychotic disorders.

The mini-review by Nieto et al. focused on recent research regarding the relationship of cognitive impairment with both Brain Derived Neurotropic Factor (BDNF) gene polymorphisms and BDNF peripheral levels in patients with various stages of psychosis. Significant relationships in clinical high-risk subjects and first-episode patients may support that BDNF change being a useful biomarker in early diagnosis. However, whether BDNF changes in the high-risk group can predict later transition to psychosis and whether pharmacological and non-pharmacological treatment can prevent such changes remains to be clarified.

Of the five empirical contributions presented on this topic, one used functional near-infrared spectroscopy, two used resting-state functional magnetic resonance imaging (MRI), and the other used structural MRI to explore potential biomarkers. Notably, Li et al. compared brain activation patterns between schizophrenia patients and healthy controls in a Chinese population during two major subtypes of verbal fluency tasks. They found that reduced left lateralization during category fluency tasks among schizophrenia patients may underlie deficient verbal fluency as a predictor of psychosis. Furthermore, Kuang et al. investigated whole-brain amplitude of low-frequency fluctuation (ALFF) changes in patients with first-episode schizophrenia and adopted a support vector regression model based on those changes to predict individual facial emotion recognition (FER) ability. Of the ALFF changes in multiple brain regions observed in the patient group, those changes especially in the limbic and occipital lobes could effectively predict fearful FER accuracy. Chen et al. measured fractional ALFF-based functional connectivity (FC) and its relevance to clinical symptoms in drug-naïve first-episode schizophrenia patients. The FC values seeded with the left orbitofrontal cortex were significantly correlated with excited symptoms in the patient group, providing important evidence regarding the pathophysiology of exciting symptoms in schizophrenia. Takahashi et al. examined the prevalence of duplicated Heschl's gyrus as a potential neurodevelopmental marker in schizophrenia patients with and without deficit syndrome. The higher prevalence of right Heschl's gyrus duplication was suggestive of prominent neurodevelopmental pathology, possibly contributing to enduring negative symptomatology in schizophrenia. Sasabayashi et al. investigated the relationship between brain gyrification patterns and subsequent relapse in patients with first-episode schizophrenia. Their findings of the higher local gyrification index (LGI) in the parieto-occipital regions in relapsed patients compared to non-relapsed patients as well as a negative correlation between inferior temporal LGI and time to first relapse may represent potential predictors that reflect relapse susceptibility in the early course of psychosis.

Accordingly, these articles accumulated many candidates for prognostic biomarkers in psychotic disorders by adopting state-of-the-art methods in various modalities. In the future, we may achieve precision medicine in psychotic disorders by collecting a large set of such biomarkers and creating a highly accurate classifier using these biological features.

## Author contributions

DS wrote the manuscript. SK, SN, and YH contributed to the writing and editing of the manuscript. All authors contributed to and approved the final manuscript.

## Funding

DS has received grants from JSPS KAKENHI (18K15509, 19H03579, 20KK0193, and 22K15745), the SENSHIN Medical Research Foundation, and the HOKURIKU BANK Grant-in-Aid for Young Scientists. SK has received grants from JSPS KAKENHI (20KK0193 and 21H02851) and AMED (JP18dm0307004 and JP19dm0207069). SN has received grants from Japan Society for the Promotion of Science (18H02755 and 22H03002), Japan Agency for Medical Research and Development (AMED), Japan Research Foundation for Clinical Pharmacology, Naito Foundation, Takeda Science Foundation, Uehara Memorial Foundation, and Daiichi Sankyo Scholarship Donation Program within the past 3 years. SN has also received research support, manuscript fees or speaker's honoraria from Dainippon Sumitomo Pharma, Meiji-Seika Pharma, Otsuka Pharmaceutical, Shionogi, and Yoshitomi Yakuhin within the past 3 years. YH has received grants from JSPS KAKENHI (JP21H02851, JP19H03579, JP18K07604, and JP19H03579), the Fund for the Promotion of Joint International Research (Fostering Joint International Research B: JP20KK0193) from the JSPS, AMED [JP20dm0207069 and GAJJ020620 (JP19dm0107124h0004)], Takeda Science Foundation, Brain Science Foundation, UBE Industries Foundation, and SIRS Research Fund Award from the Schizophrenia International Research Society.

## Conflict of interest

The authors declare that the research was conducted in the absence of any commercial or financial relationships that could be construed as a potential conflict of interest.

## Publisher's note

All claims expressed in this article are solely those of the authors and do not necessarily represent those of their affiliated organizations, or those of the publisher, the editors and the reviewers. Any product that may be evaluated in this article, or claim that may be made by its manufacturer, is not guaranteed or endorsed by the publisher.
